# A fractional order approach to modeling and simulations of the novel COVID-19

**DOI:** 10.1186/s13662-020-03141-7

**Published:** 2020-12-03

**Authors:** Isaac Owusu-Mensah, Lanre Akinyemi, Bismark Oduro, Olaniyi S. Iyiola

**Affiliations:** 1 Department of Mathematics, Ohio University, Athens, Ohio USA; 2 Department of Mathematics, Prairie View A&M University, Prairie View, Texas USA; 3 Department of Mathematics & Physical Sciences, California University of Pennsylvania, California, Pennsylvania USA; 4 Department of Science Education, University of Education, Winneba, Mampong-Ashanti Ghana

**Keywords:** COVID-19 pandemic, Transmission rate, Fractional calculus, Modeling, Simulations

## Abstract

The novel coronavirus (SARS-CoV-2), or COVID-19, has emerged and spread at fast speed globally; the disease has become an unprecedented threat to public health worldwide. It is one of the greatest public health challenges in modern times, with no proven cure or vaccine. In this paper, our focus is on a fractional order approach to modeling and simulations of the novel COVID-19. We introduce a fractional type susceptible–exposed–infected–recovered (SEIR) model to gain insight into the ongoing pandemic. Our proposed model incorporates transmission rate, testing rates, and transition rate (from asymptomatic to symptomatic population groups) for a holistic study of the coronavirus disease. The impacts of these parameters on the dynamics of the solution profiles for the disease are simulated and discussed in detail. Furthermore, across all the different parameters, the effects of the fractional order derivative are also simulated and discussed in detail. Various simulations carried out enable us gain deep insights into the dynamics of the spread of COVID-19. The simulation results confirm that fractional calculus is an appropriate tool in modeling the spread of a complex infectious disease such as the novel COVID-19. In the absence of vaccine and treatment, our analysis strongly supports the significance reduction in the transmission rate as a valuable strategy to curb the spread of the virus. Our results suggest that tracing and moving testing up has an important benefit. It reduces the number of infected individuals in the general public and thereby reduces the spread of the pandemic. Once the infected individuals are identified and isolated, the interaction between susceptible and infected individuals diminishes and transmission reduces. Furthermore, aggressive testing is also highly recommended.

## Introduction

**Figure 1:**
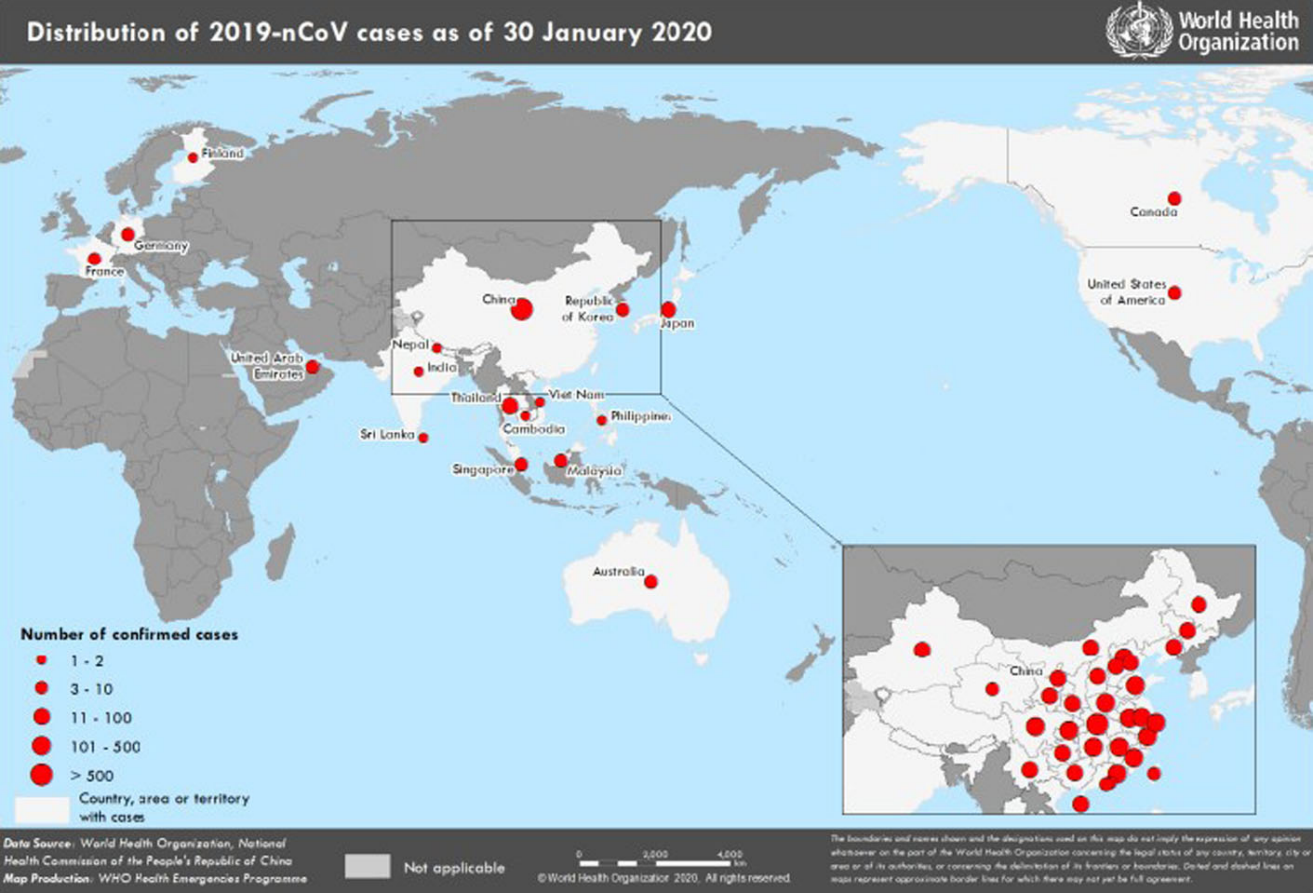
Concentration of COVID-19 cases worldwide as on January 30, 2020

**Figure 2:**
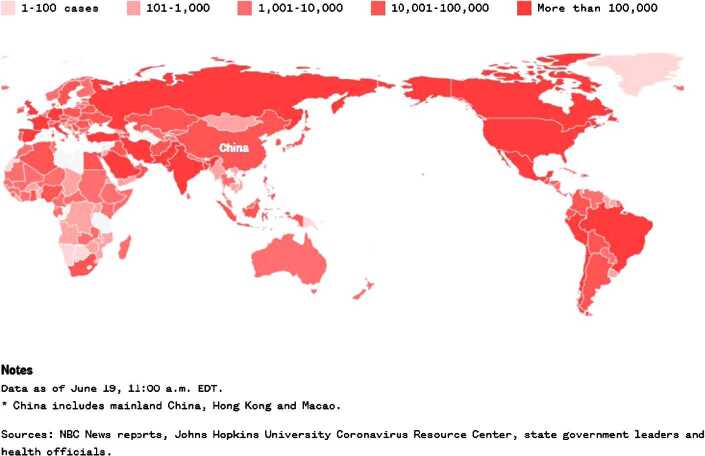
Concentration of COVID-19 cases worldwide as on June 19, 2020

Coronavirus, also known as COVID-19, has suddenly become a global pandemic that has overtaken the world by surprise, has become “cancerous” cutting across economic, politics, and social issues. The virus is said to have originated from Wuhan, a city in Hubei Province in China, estimated to occur around late December, 2019. What was thought to last for a few weeks is now considered a situation that could stay around for months or even years. COVID-19 has grown beyond the expectations of everyone. World Health Organization (WHO) declared COVID-19 a public epidemic disease in January 30, 2020, and in less than two months it was declared a pandemic with a great concern. Everyday new things are being discovered synthetically and scientifically about COVID-19. Economy has been shut down and business owners are paying big prices. As on July 10, 2020, based on the report from Johns Hopkins University & Medicine: Coronavirus Resources Center, there are over 12.4 million COVID-19 cases worldwide with over 550,000 deaths. According to CDC in the USA, the total number of cases in the USA is over 3.1 million with over 134,000 deaths. There are more than 210 countries involved. This is definitely an invisible enemy with no boundaries, and very urgent intervention is needed to understand the disease. The maps shown in Figs. 1 and 2 display the quick emergence and rapid spread of the COVID-19 with a very high level of severity.

The novel COVID-19 is an infectious disease that is caused by the severe acute respiratory syndrome virus 2 (SARS-CoV-2) belonging to the class of SARS. Though the SARS coronavirus (SARS-CoV) outbreak that happened in 2003 was fatal to 9% of infected individuals, it spread to only 26 countries and resulted in about 8000 cases [1]. The novel coronavirus outbreak, however, has become an unprecedented threat to public health worldwide. Initially, scientists know very little about the virus and struggle to provide information that is of immediate help to the health care community globally. Many things are unfolding about the disease. According to CDC, data has shown that the majority of infected patients with the COVID-19 virus have mild to moderate respiratory illness and recover without any hospitalization. However, people over 65 years and those having underlying medical problems, such as chronic lung disease, moderate to severe asthma, diabetes, serious heart conditions, immunocompromised, severe obesity (BMI 40 and above), chronic kidney disease undergoing dialysis, liver disease, and cancer, are more likely to develop serious illness and are categorized as high risk. The primary source of transmitting the novel COVID-19 is through respiratory droplets. Recently, research has shown that person to person contact by talking could spread the virus as well.

Strict guidelines, including social distancing, wearing of masks, staying at home, and local and international border closure, have been put in place to slow the spread of the virus. These pandemic restrictions have yielded positive results, reducing daily reported cases in areas with strict compliance with the protective measures. However, these intervention strategies have crippled the aviation industry, caused the shut-down of many companies, and kicked many students out of the classroom. IMF anticipates a “large global contraction in the first half of 2020,” and only the development of a vaccine or therapies can alleviate the world economy. The fear of a second wave of the pandemic has been a nightmare in the midst of countries easing restrictions or re-opening of economies.

Knowing the threat this virus poses and coupled with the fact that there is no medication or vaccination for its treatment, mathematical modeling of the spread of this virus will be one of the ways to help in curtailing and stopping the disease from plaguing the world. Mathematical and computational modeling of infectious diseases provides deeper mechanistic insights into the transmission dynamics, mitigation strategies, and prediction of the spread. Different models have been proposed for COVID-19 since the start of the pandemic, see [2–4]. Adeniyi et al. in [5] proposed and analyzed, using quantitative approach, a nonlinear mathematical model called SQIRES model to investigate the effect of healthy sanitation and awareness on the transmission dynamics of coronavirus disease (COVID-19) prevalence. Real life data from China and Italy was fitted into their model with the conclusion that good hygiene is very critical in controlling the deadly disease. Anastassopouloua et al. [6] also used the susceptible–infected–recovered–dead (SIRD) model to estimate the basic reproduction number, per day recovery, and infection rate for the data from China. SEIR model has been proposed by many to handle infectious diseases related to COVID-19, though the rate of infection is much lower compared to COVID-19. Oke et al. in [7], Okedoye et al. in [8], and Gbadamosi et al. in [9] employed SEIR model to investigate the mathematical and numerical solutions of infectious diseases models for malaria, dengue, and HIV. These authors also employed the next generation operator method to find the basic reproduction number $R_{0}$. The current work is motivated by these papers. However, integer order derivative in the classical models is not sufficient to capture the complexity nature of the virus.

It has been rigorously proven both theoretically and experimentally that fractional calculus works wonders when it comes to capturing intrinsic properties of a complex system modeling infectious diseases such as the novel COVID-19. Fractional calculus, which is a generalization of differentiation and integration of integer order, has been proposed to overcome many of the restrictions associated with integer order derivatives. Beyond biological systems, noninteger order derivatives have been successfully used to model physical phenomena in medicine, physics, image processing, optimization, electrodynamics, nanotechnology, biotechnology, engineering in general, and many more, see [10–19] and the references therein. Though every model should seek to use fractional calculus when introducing a new model, solving such a model is known to be very difficult and requires strong numerical or analytical techniques. Some of the methods used in the literature are homotopy perturbation method [20–22], Laplace analysis method [23], homotopy analysis method [24–28], Adomian decomposition method [29], differential transformation method [30], perturbation-iteration algorithm [31], iterative Shehu transform method [32], residual power series method [33–41], and q-homotopy analysis transform method in [42–45].

In the current paper, we introduce SEIR model to gain insight into the ongoing pandemic of COVID-19. The goal of this article is to propose, analyze, and simulate a compartmental COVID-19 model using fractional calculus. Our proposed model incorporates transmission rate, testing rates, and transition rate (from asymptomatic to symptomatic) for a holistic study of the infectious disease. The impacts of these parameters on the dynamics of the solution profiles for the disease are simulated and discussed in detail. Furthermore, across all the different parameters, the effects of the fractional order derivative are also simulated and discussed in great detail. Various simulations carried out enable us gain deep insights into the dynamics of the spread of COVID-19.

The rest of the paper is structured as follows: in Sect. 2, basic definitions and notations used in this present investigation are presented. Our mathematical model is formulated in Sect. 3, which accounts for the interaction between healthy individuals (susceptible) and individuals who have been either exposed or infected by the disease. Analysis of our model: existence and uniqueness of solutions, disease-free equilibrium, and basic reproduction number and sensitivity analysis are discussed in Sect. 4. The numerical simulation of the proposed model is presented in Sect. 5. Finally, Sect. 6 is devoted to summary and recommendations.

## Preliminaries

In what follows, we provide general description of fractional calculus (integral and derivative). In addition, some useful notations and established results that are needed in subsequent sections are presented. For the purpose of our study, we adopt Caputo’s fractional derivative which is most suitable for the proposed model.

### Definition 2.1

A real-valued function *φ* is said to be in the space $C_{\zeta }$, $\zeta \in \mathbb{R}$, $x>0$, if there exists a real number *p* with $p>\zeta $ such that

$$ \varphi (x)=x^{p} f(x), $$

where $f\in C[0,\infty )$ and it is said to be in the space $C_{\zeta }^{m}$ iff $l^{(m)}\in C_{\zeta }$, $m\in \mathbb{N}$.

### Definition 2.2

The Riemann–Liouville $(RL)$ fractional integral operator of order $\alpha \geq 0$ of a function $\varphi \in L^{1}(a,b)$ is given as

1 $$ \begin{aligned}& I^{\alpha }\varphi (t)= \frac{1}{\Gamma (\alpha )} \int _{0}^{t} \frac{\varphi (\xi )}{(t-\xi )^{1-\alpha }}\,d\xi ,\quad t>0, \alpha >0, \\ &I^{0}\varphi (t)=c(t). \end{aligned} $$

The notation Γ is the well-known gamma function.

### Definition 2.3

Fractional differential operator in the sense of Caputo is defined in general for $\alpha >0$ and $t>0$ as follows [46]:

2 $$\begin{aligned} \mathcal{D}^{\alpha }\varphi (t)=I^{n-\alpha }D^{n} \varphi (t)=\textstyle\begin{cases} \frac{1}{\Gamma (n-\alpha )}\int _{0}^{t} \frac{\varphi ^{(n)}(\xi )}{(t-\xi )^{\alpha +1-n}}\,d\xi ,& \text{if } n-1< \alpha \leq n \in \mathbb{N}, \\ \frac{d^{n}\varphi (t)}{dt^{n}},& \alpha =n\in \mathbb{N}. \end{cases}\displaystyle \end{aligned}$$

Caputo fractional differential operator naturally attracts classical initial conditions (not integral type initial conditions) that is suitable for our model.

### Lemma 2.4

*Let* $t\in (a,b]$. *Then*

3 $$ \bigl[I_{a}^{\alpha }(t-a)^{\beta } \bigr](t)= \frac{\Gamma (\beta +1)}{\Gamma (\beta +\alpha +1)}(t-a)^{\beta + \alpha },\quad \alpha \geqslant 0, \beta >0. $$

For easy use, we denote $Z(t)=(S(t),E(t), I_{1}(t),I_{2}(t),C(t),R(t))$ and $\mathbb{R}^{6}_{+} = \{ Z\in \mathbb{R}^{6}: Z\geq 0 \} $.

### Lemma 2.5

([47])

*For* $0<\alpha \leq 1$, *let* $\varphi (t)\in C[a,b]$ *and* $\mathcal{D}^{\alpha }\varphi (t)\in (a,b]$. *Then*

4 $$ \varphi (t)=\varphi (a)+\frac{1}{\Gamma (\alpha )}\mathcal{D}^{\alpha }\varphi (\eta ) (t-a)^{\alpha },\quad 0\leq \eta \leq t, \forall t\in (a,b]. $$

Lemma 2.5 is named the generalized mean value theorem.

## Model formulation

**Figure 3:**
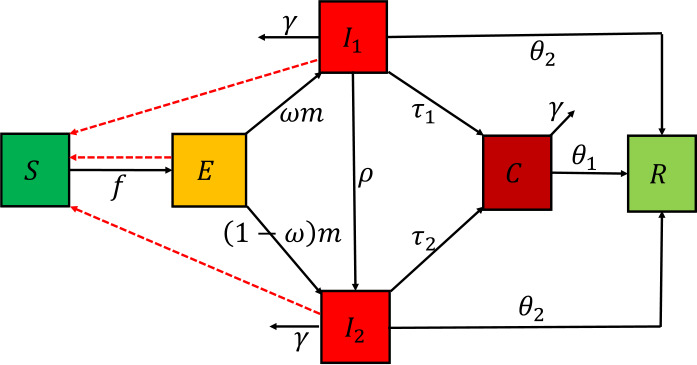
Flow diagram of the model. $f=\beta S(E+I_{1}+I_{2})$

We develop a compartmental model based on the development and epidemiological characteristics of COVID-19. The population at time *t* is divided into susceptible, exposed, asymptomatic, symptomatic, confirmed, and recovered classes. These sub-populations are denoted by $S(t)$, $E(t)$, $I_{1}(t)$, $I_{2}(t)$, $C(t)$, and $R(t)$ respectively. There are some people infected with COVID-19 and do not develop symptoms but are still able to pass the disease to others, these individuals spread it silently. The CDC in [48] estimated that $35\%-40\%$ of coronavirus patients do not have symptoms and about 40% of COVID-19 transmission occurs before people feel sick. With these in mind, we assume that exposed, asymptomatic, and symptomatic individuals are able to transmit the disease to the general public. We further assume that individuals who have been clinically tested and confirmed positive for the COVID-19 disease are either under self-quarantine or hospitalized and are unable to transmit the disease to the general public. The transmission rate is given by *β* and the incubation period is given by $1/m$. After the incubation period, individuals from *E* class move to $I_{2}$ at the rate $(1-w)m$, where *w* is the fraction of individuals who are asymptomatic patients. The dynamics explained in this subsection are displayed in Fig. 3, also see Table 1 for the meaning of the remaining parameters. A fractional order model satisfying the above description is of the form

5 $$\begin{aligned}& \begin{gathered} \frac{d^{\alpha }S}{dt^{\alpha }}=-\beta (E+I_{1}+I_{2})S, \\ \frac{d^{\alpha }E}{dt^{\alpha }}=\beta (E+I_{1}+I_{2})S- mE, \\ \frac{d^{\alpha }I_{1}}{dt^{\alpha }}= \omega m E- (\tau _{1}+\rho + \theta _{2} +\gamma ) I_{1}, \\ \frac{d^{\alpha }I_{2}}{dt^{\alpha }}= (1-\omega ) m E+\rho I_{1}-(\tau _{2} +\theta _{2} +\gamma ) I_{2}, \\ \frac{d^{\alpha }C}{dt^{\alpha }} = \tau _{1} I_{1}+\tau _{2}I_{2}-( \theta _{1}+\gamma ) C, \\ \frac{d^{\alpha }R}{dt^{\alpha }}= \theta _{2}(I_{1}+I_{2})+ \theta _{1}C, \end{gathered} \end{aligned}$$

and initial points are

6 $$\begin{aligned} \begin{gathered} S(0)=S_{0},\qquad E(0)=E_{0},\qquad I_{1}(0)=I_{(1,0)},\qquad I_{2}(0)=I_{(2,0)},\\ C(0)=C_{0},\qquad R(0)=R_{0}, \end{gathered} \end{aligned}$$

where $0<\alpha \leq 1$ and $\frac{d^{\alpha }}{dt^{\alpha }}$ is the Caputo fractional derivative of order *α*. All other parameters are described in Table 1.

**Table 1:** Parameters of the disease model and their meanings

Parameter	Range (Sources)	Default value
*β* (transmission rate)	2.1011 × 10^−8^–9.11 × 10^−8^ [49, 50]	3.511 × 10^−8^ day^−1^
1/*m* (inverse incubation period)	1/14–1/2 day^−1^	1/7 day^−1^
*ω* (fraction of infected individuals that do not show symptoms)	0.35–0.4 [48]	0.37
$\tau _{1}$ (progression rate of asymptomatic individuals to confirmed, depends on contact tracing and testing)	1/20–1/5 day^−1^ [50, 51]	1/15 day^−1^
$\tau _{2}$ (progression rate of symptomatic individuals to confirmed, depends on contact tracing and testing)	1/5–1 day^−1^ [50, 51]	1/3 day^−1^
$\theta _{1}$ (recovery rate, confirmed)	0.11624 day^−1^ [49]	0.11624 day^−1^
$\theta _{2}$ (natural recovery rate)	0.13798 day^−1^ [49]	0.13798 day^−1^
*γ* (disease mortality rate)	1.7826 × 10^−5^ [49, 50]	1.7826 × 10^−5^
*ρ* (transition rate from asymptomatic to symptomatic)	assumed	0.2
*S*(0) (initial value of the susceptible)	[50]	11,081,000
*E*(0) (initial value of the expose)	[50]	399
$I_{1}(0)$ (initial value asymptomatic)	[50]	28
$I_{2}(0)$ (initial value of symptomatic)	[50]	54
*C*(0) (initial value of confirmed)	[50]	41
*R*(0) (initial value of recovered)	[50]	12

## Model analysis

In this section, we establish the existence and uniqueness of solutions to our model in Eqs. (5)–(6), set up the disease free equilibrium and an expression for the basic reproduction number of our model. We further carry out a sensitive analysis to help identify parameters that need to be targeted for the design of control strategies.

### Existence and uniqueness of solutions

The results on the existence and uniqueness of solutions to the system in Eqs. (5)–(6) are considered. In addition, we show that the domain is positively invariant.

#### Lemma 4.1

*For* $0<\alpha \leq 1$, *let* $w\in C[0,b]$ *and* $\mathcal{D}^{\alpha }w\in (0,b]$. *Then*

(i) *the function* *w* *is nondecreasing if* $\mathcal{D}^{\alpha }w(t)\geq 0$, $\forall t\in (0,b)$.
(ii) *the function* *w* *is nonincreasing if* $\mathcal{D}^{\alpha }w(t)\leq 0$, $\forall t\in [0,b]$.

#### Proof

The proof is a direct consequence of Lemma 2.5. □

#### Theorem 4.2

*The IVP for the generalized time*-*fractional COVID*-19 *model given in Eqs*. (5)*–*(6) *has unique solution in* $\mathbb{R}^{6}_{+}$.

#### Proof

By Remark 3.2 in [52] together with Lemma 4.1, the existence and uniqueness of solution in $(0,\infty )$ are obtained. In addition, we obtain the following estimates, noting that $\omega <1$:

$$\begin{aligned}& \frac{d^{\alpha }S}{dt^{\alpha }}\biggm|_{S=0} = 0, \\& \frac{d^{\alpha }E}{dt^{\alpha }}\biggm|_{E=0} = \beta (I_{1}+I_{2})S \geq 0, \\& \frac{d^{\alpha }I_{1}}{dt^{\alpha }}\biggm|_{I_{1}=0} = \omega m E \geq 0, \\& \frac{d^{\alpha }I_{2}}{dt^{\alpha }}\biggm|_{I_{2}=0} = (1-\omega ) m E+ \rho I_{1} \geq 0, \\& \frac{d^{\alpha }C}{dt^{\alpha }}\biggm|_{C=0} = \tau _{1} I_{1}+ \tau _{2}I_{2} \geq 0, \\& \frac{d^{\alpha }R}{dt^{\alpha }}\biggm|_{R=0} = \theta _{2}(I_{1}+I_{2})+ \theta _{1}C \geq 0 \end{aligned}$$

on each hyperplane bounding the nonnegative orthant. Hence the domain $\mathbb{R}^{6}_{+}$ is positively invariant. This completes the proof. □

### Disease-free equilibrium and basic reproduction number

In the absence of COVID-19, we have $E=I_{1}=I_{2}=C=0$ and at equilibrium

7 $$\begin{aligned} S^{*}=N^{*}=S(0) \quad \text{and} \quad R^{*}=0. \end{aligned}$$

There exists a disease-free equilibrium of system Eq. (5) given by

8 $$\begin{aligned} \aleph _{0}= \bigl(S(0),0,0,0,0,0 \bigr). \end{aligned}$$

Note that the point $\aleph _{1}= (0,0,0,0,0,N(0) )$ is also a disease-free equilibrium. At this equilibrium, there are no susceptible individuals and surviving infested individuals eventually recover.

The basic reproductive number (${\mathcal{R}}_{0}$) is defined in [53] as *the average number of secondary infections that occur when one infected individual is introduced into a completely susceptible population.* It is one of the most significant thresholds when studying infectious disease models; it quantifies the intensity of an outbreak of disease. It also plays an important role in evaluating control strategies. Following the next generation operator method and notation in [54–56], we compute ${\mathcal{R}}_{0}$ and explore the local stability of $\aleph _{0}$. The method is defined as the dominant eigenvalue (spectral radius) of the matrix $FV^{-1}$, where *F* and $V^{-1}$ are matrices associated with the vector $F_{i}$ (of new infections) and the vector $V_{i}$ (of the transfer of individuals between classes) respectively. Thus,

$$ F_{i}= \begin{bmatrix} F_{E} \\ F_{I_{1}} \\ F_{I_{2}} \\ F_{C} \end{bmatrix} = \begin{bmatrix} &\beta (E+I_{1}+I_{2})S \\ &0 \\ &0 \\ &0 \end{bmatrix} $$

and

$$ V_{i}= \begin{bmatrix} V_{E} \\ V_{I_{1}} \\ V_{I_{2}} \\ V_{C} \end{bmatrix} = \begin{bmatrix} &mE \\ &-\omega mE+(\tau _{1}+\rho +\theta _{2} +\gamma )I_{1} \\ &-(1-\omega ) mE-\rho I_{1}+(\tau _{2} +\theta _{2} +\gamma ) I_{2} \\ &-\tau _{1} I_{1}-\tau _{2}I_{2}+(\theta _{1}+\gamma ) C \end{bmatrix} . $$

Therefore

$$ F= \begin{bmatrix} \beta S^{*} &\beta S^{*}&\beta S^{*}&0 \\ 0 &0&0&0 \\ 0 &0&0&0 \\ 0 &0&0&0 \end{bmatrix} $$

and

$$ V= \begin{bmatrix} m &0&0&0 \\ -\omega m &\tau _{1}+\rho +\theta _{2} +\gamma &0&0 \\ -(1-\omega ) m &-\rho &\tau _{2} +\theta _{2} +\gamma &0 \\ 0 &-\tau _{1}&-\tau _{2}&\theta _{1}+\gamma \end{bmatrix} . $$

The basic reproduction number (${\mathcal{R}}_{0}$) is given by

9 $$\begin{aligned} {\mathcal{R}}_{0}=\beta S(0) \biggl( \frac{1}{m}+ \frac{\gamma +\theta _{2}+\rho +(1-\omega )\tau _{1}+\omega \tau _{2}}{ (\gamma +\theta _{2}+\rho +\tau _{1} ) (\gamma +\theta _{2}+\tau _{2} )} \biggr). \end{aligned}$$

The results below follow from Theorem 2 in [55].

#### Theorem 4.3

*The disease*-*free equilibrium of the model Eq*. (5), *given by* $\aleph _{0}$, *is locally*-*asymptotically stable* (*LAS*) *if* ${\mathcal{R}}_{0}<1$, *and unstable if* ${\mathcal{R}}_{0}>1$.

The public health implication of Lemma 4.3 is that the infected population can be eliminated or controlled if ${\mathcal{R}}_{0}<1$.

#### Theorem 4.4

*Let* $\rho =0$. *Then the disease*-*free equilibrium* $(S(0),0,0,0,0,0 )$ *of the system in Eq*. (6) *is globally asymptotically stable if* ${\mathcal{R}}_{0}<1$.

#### Proof

Consider the Lyapunov function $L=(S,E,I_{1},I_{2},C,R):\mathbb{R}^{6}_{+}$ defined as

$$ L=BE+I_{1}+B_{1}I_{2} $$

for some constants $B,B_{1} > 0$ that will be determined later. The time derivative of *L* is

10 $$\begin{aligned} \frac{d^{\alpha }L}{dt^{\alpha }} =& B\frac{d^{\alpha }E}{dt^{\alpha }} + \frac{d^{\alpha }I_{1}}{dt^{\alpha }}+ B_{1} \frac{d^{\alpha }I_{2}}{dt^{\alpha }} \\ =& B \bigl[\beta (E + I_{1} + I_{2})S - mE \bigr] + \bigl[\omega m E - (\tau _{1} + \theta _{2} + \gamma ) I_{1} \bigr] \\ &{}+ B_{1} \bigl[(1- \omega ) m E -( \tau _{2} + \theta _{2} + \gamma ) I_{2} \bigr] \\ \leq & B \bigl[\beta (E+I_{1}+I_{2})S(0)- mE \bigr]+ \bigl[\omega m E- (\tau _{1} +\theta _{2} + \gamma ) I_{1} \bigr] \\ &{} + B_{1} \bigl[(1-\omega )m E-(\tau _{2} + \theta _{2} +\gamma ) I_{2} \bigr] \\ =& B \bigl(\beta ES(0)- mE \bigr) + \bigl(\omega + B_{1}(1 - \omega ) \bigr)mE + B\beta (I_{1} + I_{2})S(0) \\ &{} - (\tau _{1} + \theta _{2} + \gamma ) I_{1} - B_{1}(\tau _{2} + \theta _{2} +\gamma ) I_{2} \\ =& Bm \biggl(\frac{\beta S(0)}{m}- 1 \biggr)E+ \bigl(\omega +B_{1}(1- \omega ) \bigr)mE+B\beta (I_{1}+I_{2})S(0) \\ &{} -(\tau _{1} + \theta _{2} +\gamma ) I_{1} - B_{1}(\tau _{2} + \theta _{2} +\gamma ) I_{2} \\ =& Bm \biggl(\frac{\beta S(0)}{m}+\beta S(0) \frac{\gamma +\theta _{2}+(1-\omega )\tau _{1}+\omega \tau _{2}}{ (\gamma +\theta _{2}+\tau _{1} ) (\gamma +\theta _{2}+\tau _{2} )}- 1 \biggr)E \\ &{} -B\beta S(0)mE \frac{\gamma +\theta _{2}+(1-\omega )\tau _{1}+\omega \tau _{2}}{ (\gamma +\theta _{2}+\tau _{1} ) (\gamma +\theta _{2}+\tau _{2} )} \\ & {}+ \bigl(\omega + B_{1}(1-\omega ) \bigr)mE+B\beta (I_{1} + I_{2})S(0) - (\tau _{1} +\theta _{2} +\gamma ) I_{1}-B_{1}(\tau _{2} +\theta _{2} +\gamma ) I_{2} \\ =& Bm ({\mathcal{R}}_{0} - 1 )E- \biggl[B\beta S(0) \frac{\gamma +\theta _{2} + (1 - \omega )\tau _{1} + \omega \tau _{2}}{ (\gamma +\theta _{2} + \tau _{1} ) (\gamma + \theta _{2} + \tau _{2} )}- \bigl(\omega + B_{1}(1-\omega ) \bigr) \biggr]mE \\ & {}+ B\beta (I_{1} + I_{2})S(0) - ( \tau _{1} + \theta _{2} + \gamma ) I_{1} -B_{1}(\tau _{2} + \theta _{2} + \gamma ) I_{2}. \end{aligned}$$

Choose $B=\frac{\tau _{1} +\theta _{2} +\gamma }{\beta S(0)}$ and $B_{1}= \frac{\tau _{1} +\theta _{2} +\gamma }{\tau _{2} +\theta _{2} +\gamma }$ so that Eq. (10) reduces to

$$\begin{aligned} \frac{d^{\alpha }L}{dt^{\alpha }} \leq & \frac{\tau _{1} +\theta _{2} +\gamma }{\beta S(0)}m ({ \mathcal{R}}_{0}- 1 )E+\frac{(\tau _{1} +\theta _{2} +\gamma )}{\beta S(0)}\beta (I_{1}+I_{2})S(0) - (\tau _{1} +\theta _{2} +\gamma ) I_{1} \\ &{} - \frac{(\tau _{1} + \theta _{2} + \gamma )}{(\tau _{2} + \theta _{2} +\gamma )}( \tau _{2} + \theta _{2} + \gamma ) I_{2} \\ & {}- \biggl[\frac{\tau _{1} + \theta _{2} + \gamma }{\beta S(0)}\beta S(0) \frac{\gamma + \theta _{2} + (1 - \omega )\tau _{1} + \omega \tau _{2}}{ (\gamma + \theta _{2} + \tau _{1} ) (\gamma + \theta _{2} +\tau _{2} )}\\ &{} - \biggl( \omega + \frac{\tau _{1} + \theta _{2} + \gamma }{\tau _{2} + \theta _{2} + \gamma }(1 - \omega ) \biggr) \biggr]mE \\ =& \frac{\tau _{1} +\theta _{2} + \gamma }{\beta S(0)}m ({\mathcal{R}}_{0} - 1 )E\\ &{} - \biggl[ \frac{\gamma + \theta _{2} + \tau _{1} + \omega (\tau _{2} - \tau _{1})}{ (\gamma + \theta _{2} + \tau _{2} )} - \biggl(\omega + \frac{\tau _{1} + \theta _{2} + \gamma }{\tau _{2} +\theta _{2} + \gamma }(1 - \omega ) \biggr) \biggr]mE \\ =& \frac{\tau _{1} +\theta _{2} +\gamma }{\beta S(0)}m ({\mathcal{R}}_{0}- 1 )E< 0 \end{aligned}$$

if ${\mathcal{R}}_{0}<1$. Note that $\frac{d^{\alpha }L}{dt^{\alpha }}=0$ if and only if $E=0$. By the generalized LaSalle invariance principle [57], all trajectories that start in $\mathbb{R}^{6}_{+}$ approach $\aleph _{0}$ as $t \rightarrow \infty $. □

### Sensitivity analysis

**Figure 4:**
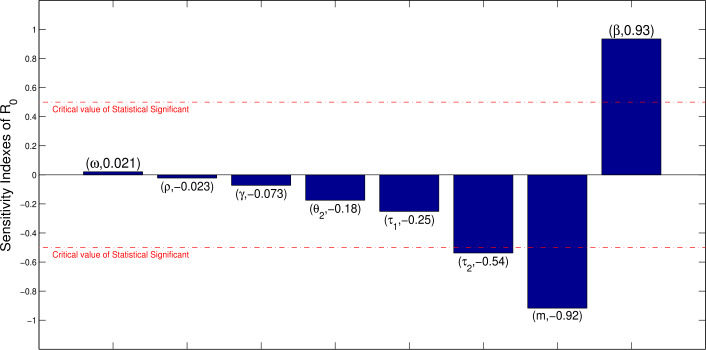
Sensitivity analysis

We carried out an uncertainty and sensitivity analysis using the Latin hypercube sampling (LHS), a statistical scheme for generating a sample of likely parameter values from a multidimensional distribution, and partial rank correlation coefficients (PRCCs), “a robust sensitivity measure for nonlinear but monotonic relationships between input and output, as long as little to no correlation exists between the inputs” [58–61], to identify model parameters that have most influence on the threshold ${\mathcal{R}}_{0}$ and the COVID-19 transmission. Sensitivity analysis is useful and can help to identify parameters that need to be targeted in designing control strategies. The index measures the relative change in ${\mathcal{R}}_{0}$ with respect to the relative change in the parameters [62, 63]. The parameters considered in the PRCCs analysis include transmission rates (*β*), transition rates($\tau _{1}$, $\tau _{2}$, *ρ*, *m*, *ω*), recovery rates of virus ($\theta _{1}$, $\theta _{2}$), and disease mortality rate (*γ*). A parameter with large PRCC (greater than +0.50 or less than −0.50) is assumed to be sensitive. Figure 4 shows the PRCCs of the parameters with ${\mathcal{R}}_{0}$ as the response function.

From the results, ${\mathcal{R}}_{0}$ is more sensitive to $\tau _{2}$, *m*, and *β* in increasing order, among the parameters considered in the determination of basic reproduction number. Parameters $\tau _{2}$, *m* have a negative impact on ${\mathcal{R}}_{0}$, meaning that an increase in these parameters will reduce ${\mathcal{R}}_{0}$, while *β* has a positive impact, and reducing the value of this parameter will reduce ${\mathcal{R}}_{0}$.

The following can be inferred from the sensitive analysis:

1. Interventions that reduce the value of the transmission (contact) rate *β* could be effective control measures to stop the spread of the coronavirus.
2. An increase in the tracing and testing of individuals that have had contact with infected persons could help in the fight against the virus, since this will increase the progression rate $\tau _{2}$. They can then be quarantined or put in isolation so that they will not affect other susceptible individuals.

It is well known that when the reproduction number ${\mathcal{R}}_{0}$ is below unity, then an outbreak will die out. Based on Eq. (9) and the parameter values in Table 1, ${\mathcal{R}}_{0}=3.7501$. This estimated threshold is close to the lower bound of ${\mathcal{R}}_{0}$ estimated in [64]. We investigated scenarios under which ${\mathcal{R}}_{0}$ captured in this study could fall below 1.

Based on Table 2, we have the following:

(i) In Scenario 1, the basic reproductive number dropping below unity is achievable if there is at least 74% reduction in the baseline of the transmission parameter. This could crush the spread of the pandemic. Thus, it is important to adhere to face mask use, social distancing, washing hands protocols to contain the outbreak.
(ii) In Scenario 2, the result suggests that mass testing alone is not enough to curb the spread. This shows that a 100% increase in both testing rates is not able to reduce the reproduction number below unity.
(iii) For the case in Scenario 3, combination of at least 71% reduction in the baseline of the transmission parameter and a 100% increase in both testing rates produces the desirable reproduction number.

**Table 2:** Assessing the basic reproduction number, ${\mathcal{R}}_{0}$

Parameter	Baseline as in Table 1	Scenario 1	Scenario 2	Scenario 3
*β*	3.511 × 10^−8^	74% reduction	–	71% reduction
1/*m*	1/7	–	–	–
*ω*	0.37	–	–	–
$\tau _{1}$	1/15	–	100% increase	100% increase
$\tau _{2}$	1/3	–	100% increase	100% increase
$\theta _{1}$	0.11624	–		
$\theta _{2}$	0.13798	–	–	–
*γ*	1.7826 × 10^−5^	–	–	–
*ρ*	0.2	–	–	–
${\mathcal{R}}_{0}$	3.7501	0.9750	3.4093	0.9887

## Numerical solutions and analysis

We give a brief introduction of the idea behind the method employed to solve the proposed COVID-19 model of fractional order type given in Eqs. (5)–(6). Also, many numerical simulations are carried out to investigate the effects of various parameters including the fractional order *α* and the sensitive parameters identified in the previous section.

### Generalized form of Adams–Bashforth–Moulton algorithm

First, the Adams–Bashforth–Moulton method, also known as the predictor–corrector method, is introduced for integer order differential equations. We refer the reader for full detail analysis of the convergence, accuracy, and stability of the method in [65–67]. Consider an IVP fractional differential equation

11 $$\begin{aligned} \begin{aligned}& \mathcal{D}_{t}^{\alpha }v(t) = f\bigl(t,v(t)\bigr) \\ &v^{(k)}(0)= v_{0}^{k},\quad k = 0,1,2, \ldots ,m-1 \end{aligned} \end{aligned}$$

with $\alpha >0$, $m=\lceil \alpha \rceil $ and $v_{0}^{k}$, $k = 0,1,2,\ldots ,m-1$ are given real numbers. Clearly a function $v(t)$, continuous, is a solution of IVP Eq. (11) if and only if it solves the following Volterra integral equation:

12 $$ v(t) = \sum_{k=0}^{\lceil \alpha \rceil -1} \frac{t^{k}}{k!}v_{0}^{k} + \frac{1}{\Gamma (\alpha )} \int _{0}^{t}(t-\sigma )^{\alpha -1}g\bigl( \sigma ,\phi (\sigma )\bigr)\,d\sigma . $$

Next, a generalized form of Adams–Bashforth–Moulton method for fractional order (see details in [66]) is introduced. The same technique used to derive one-step Adams-Bashforth-Moulton method for integer order, $\alpha = 1$, is employed to derive the generalized case with the following basic assumptions:

- Uniform grid with $t_{n}=nh$, $n=0,1,2,\ldots ,N$, $N\in \mathbb{N}$, $h>0$ is the step size.
- We first compute the approximation $v_{h}(t_{i})\approx v(t_{i})$, $i=1,2,\ldots ,n$, then find the approximation $v_{h}(t_{n+1})$ using Eq. (12).

For the corrector, product trapezoidal quadrature formula is employed to compute the integral in Eq. (12) with nodes $t_{i}$, $i=0,1,2,\ldots ,n+1$. However, for the predictor, a product rectangle rule is used. Combining these two, the scheme for the solution of fractional order differential Eq. (11) called generalized Adams–Bashforth–Moulton method is given as follows:

13 $$\begin{aligned} v_{h}(t_{n+1}) =& \sum _{k=0}^{\lceil \alpha \rceil -1} \frac{t_{n+1}^{k}}{k!}v_{0}^{k} \\ &{} + \frac{h^{\alpha }}{\Gamma (\alpha +2)}g \bigl(t_{n+1},v_{h}^{p}(t_{n+1}) \bigr) + \frac{h^{\alpha }}{\Gamma (\alpha +2)}\sum_{i=0}^{n} \varepsilon _{i,n+1} f \bigl(t_{i},v_{h}(t_{i}) \bigr), \end{aligned}$$

14 $$\begin{aligned} v_{h}^{p}(t_{n+1}) =& \sum _{k=0}^{\lceil \alpha \rceil -1} \frac{t_{n+1}^{k}}{k!}v_{0}^{k} + \frac{1}{\Gamma (\alpha )}\sum_{i=0}^{n} \omega _{i,n+1} f \bigl(t_{i},v_{h}(t_{i}) \bigr), \end{aligned}$$

where

15 $$\begin{aligned} \varepsilon _{i,n+1} = \textstyle\begin{cases} 1&\text{if }i=n+1, \\ n^{\alpha +1}-(n-\alpha )(n+1)^{\alpha }& \text{if } i=0, \\ (n+2-i)^{\alpha +1}+(n-i)^{\alpha +1}-2(n+1-i)^{\alpha +1}&\text{if }1\leq i\leq n, \end{cases}\displaystyle \end{aligned}$$

and

16 $$\begin{aligned} \omega _{i,n+1} = \frac{h^{\alpha }}{\alpha } \bigl[(n+1-i)^{\alpha }-(n-i)^{\alpha } \bigr]. \end{aligned}$$

Some numerical analysis details and the MATLAB subroutine fde12 implementation of the method above can be found in [68]. The subroutine is in our numerical simulation.

### Numerical results and analysis

We present the numerical simulations of the proposed time-fractional order COVID-19 system to study the spread and containment strategies of the coronavirus infection. The parameter values (both baseline and range) used for the simulations were taken from literature as given in Table 1, unless otherwise stated. It is of interest to see various intrinsic properties of the COVID-19 model that could be shown using fractional derivative ($0 < \alpha < 1$) in time, compared to classical order $\alpha =1$. First, we simulate with different values of fractional order *α* with fixed values of the model parameters (baseline values). Next, we examine the effects of changing the sensitive parameters obtained in Sect. 4.3. It is important to further examine the impact of these key parameters on the solution profiles for crucial decisions, given different values of fractional order as well.

#### Effect of time-fractional order on the time-line of the virus infection

**Figure 5:**
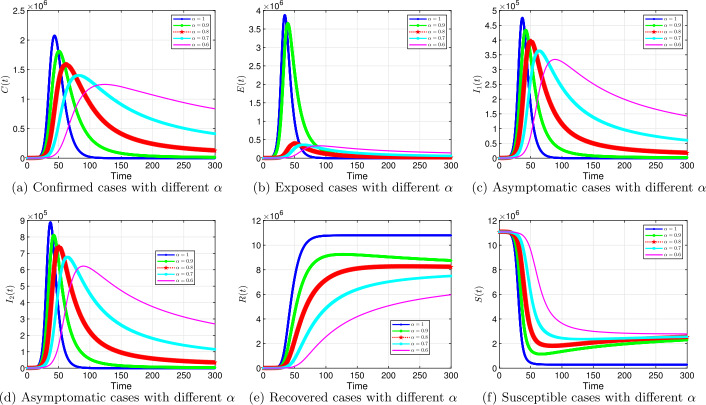
Solution profiles for the COVID-19 model with different *α*

Using the baseline values of the parameters, we simulate COVID-19 model proposed for different values of fractional order *α*. The epidemic trajectories for the proposed fractional order COVID-19 model are provided in Fig. 5 for different values of *α*. The effects of fractional orders are distinctive; the solution curves for $0<\alpha <1$ show delay in the epidemic peak and flatten faster, see Fig. 5 (a), (c), and (d). These observations are known to occur in epidemic models with intervention [69]. The impacts of *α* is even more pronounced for smaller orders; for example, compare $\alpha =0.9$ and $\alpha =0.6$ in Fig. 5 (c). While we observe significant reduction in the number of infected individuals for smaller fractional orders, the number of susceptible individuals climb up as shown in Fig. 5 (f). It should be mentioned here that hospitalization will not be overwhelmed for the cases of $\alpha <1$ due to flattening of the curve. This situation has been observed by Ecuador, Pakistan, United Kingdom, and Chile as reported by John Hopkins University and Medicine on July 1, 2020. However, countries like United States, Brazil, Mexico, and India have a similar trend as we have for the case when $\alpha =1$. The peak rose very quickly and overwhelmed the hospital facilities.

#### Transmission rate *β*

**Figure 6:**
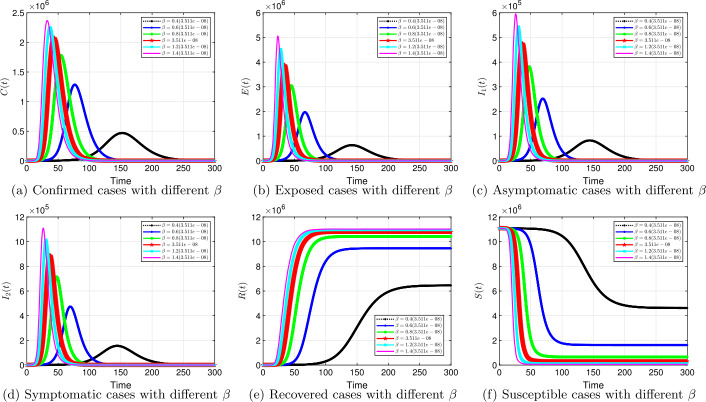
Solution profiles for the COVID-19 model with different *β* when $\alpha =1$

**Figure 7:**
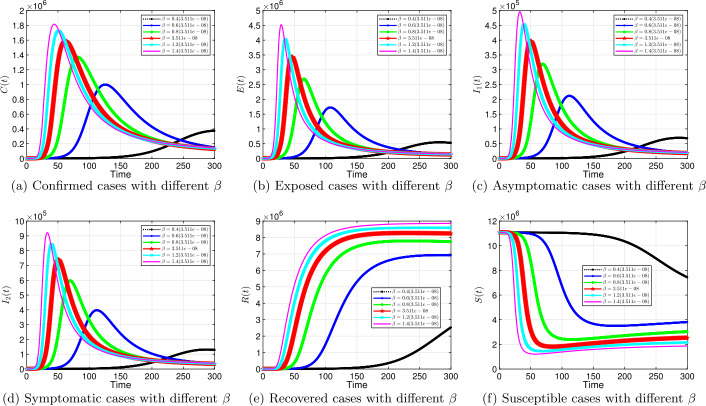
Solution profiles for the COVID-19 model with different *β* when $\alpha =0.9$

**Figure 8:**
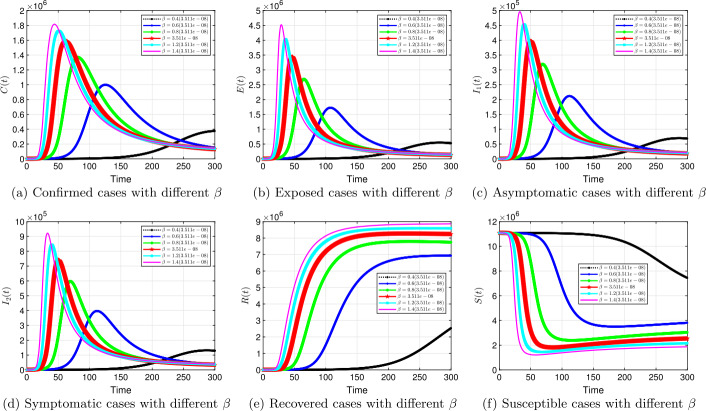
Solution profiles for the COVID-19 model with different *β* when $\alpha =0.8$

**Figure 9:**
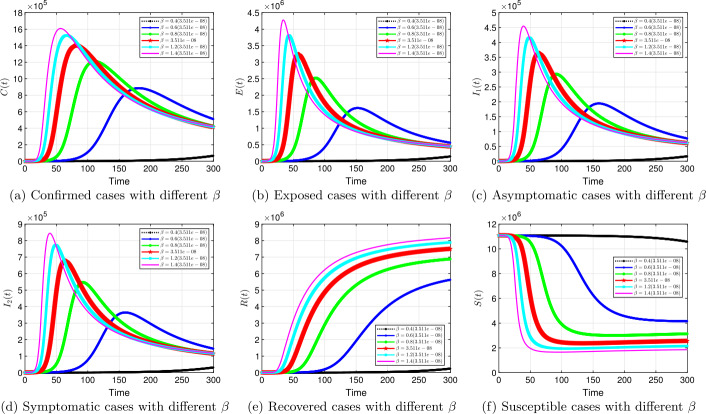
Solution profiles for the COVID-19 model with different *β* when $\alpha =0.7$

Transmission rate *β* is the focus here. We investigate the effects of the transmission rate in the dynamics of the spread of COVID-19. Figures 6 to 9 show results for the effects of different transmission rates to maneuver this virus down. Similar to the effects of fractional order, a smaller transmission rate delays the peak significantly and reduces the number of infected cases, as displayed in Fig. 6 (a), (c), and (d); Fig. 7 (a), (c), and (d); Fig. 8 (a), (c), and (d), and Fig. 9 (a), (c), and (d). For example, in Fig. 6 (c), at a baseline $\beta =3.511e-08$ with $\alpha =1$, approximately $4.8 \times 10^{5}$ asymptomatic cases are observed. A 60% reduction in the baseline produces less than $100,000$ cases (the black curve). The influence of the transmission rate on the dynamics is robust for the other fractional orders, see Figs. 7 to 9. Reducing the transmission parameter leads to substantial decrease in infected cases. These results are consistent with the results in Table 2 (Scenario 1), where a reduction of the transmission rates by 74%, with other parameters fixed, keeps ${\mathcal{R}}_{0}$ less than 1. As at now, there is no vaccine, non-pharmaceutical interventions are recommended for reducing the rate of transmission and the spread of the SARS-CoV-2. It should be noted that there is a significant reduction in infected cases, when controlling the transmission rate with lower fractional order *α*. Our results show that our proposed fractional model captures characteristics of many countries by changing the value of *α*.

#### Progression rates $\tau _{1}$, $\tau _{2}$

**Figure 10:**
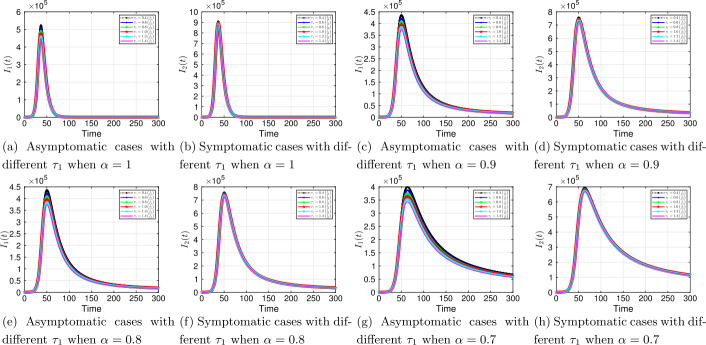
Solution profiles for the COVID-19 model with different $\tau _{1}$ across different fractional order *α*

**Figure 11:**
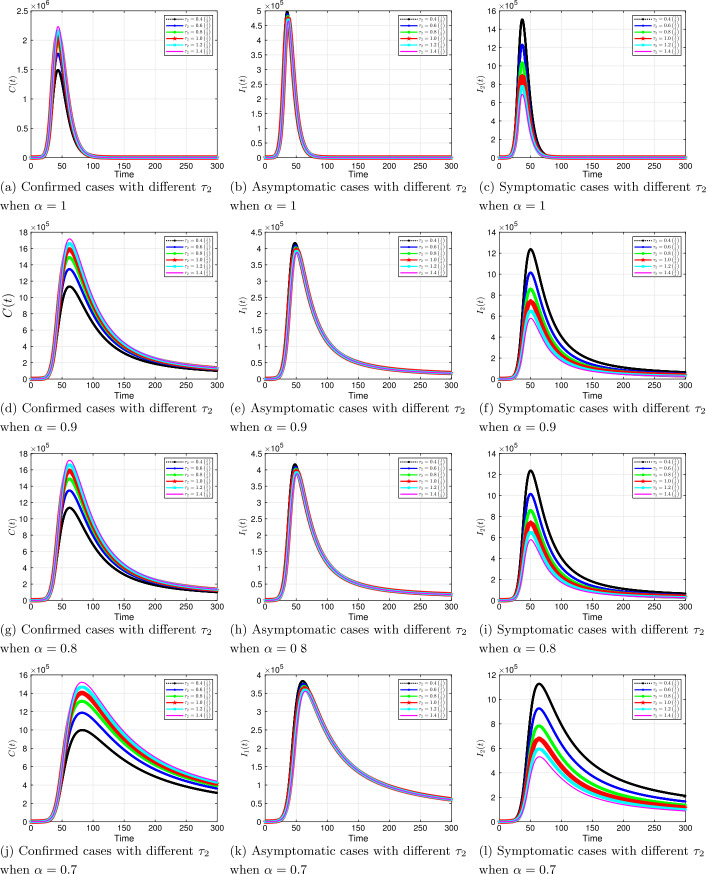
Solution profiles for the COVID-19 model with different $\tau _{2}$ across different fractional order *α*

Next, we vary the progression rate for symptomatic $\tau _{1}$ and asymptomatic $\tau _{2}$ parameters to examine their influence on the dynamics of the COVID-19 system. The results are shown in Figs. 10 and 11. We observe from the results above that increasing (decreasing) these parameters by a given percentage always increases (decreases) the number of confirmed positive coronavirus cases. The effects are also felt both in the asymptomatic and symptomatic classes; increasing (decreasing) $\tau _{1}$ and $\tau _{2}$ decreases (increases) individuals in these classes. For example, at time $t=50$, increasing the baseline $\tau _{2}=1/3$ by 40% decreases the infection in the symptomatic compartment from about $7.5 \times 10^{5}$ to $5.7 \times 10^{5}$ individuals. It is important to point out that these parameters are associated with contact tracing and testing of individuals. As we observed in Table 2 scenario 2, increasing only the progression rates through rigorous contact tracing and testing is not enough to curb the virus since ${\mathcal{R}}_{0}$ is greater than one.

#### Transition rate, *ρ*: from asymptomatic to symptomatic

Lastly, we examine the epidemic dynamics for different transition rate from asymptomatic to symptomatic *ρ*. Our focus here is on the potential for infectious individuals who do not have symptoms to eventually develop symptoms.

**Figure 12:**
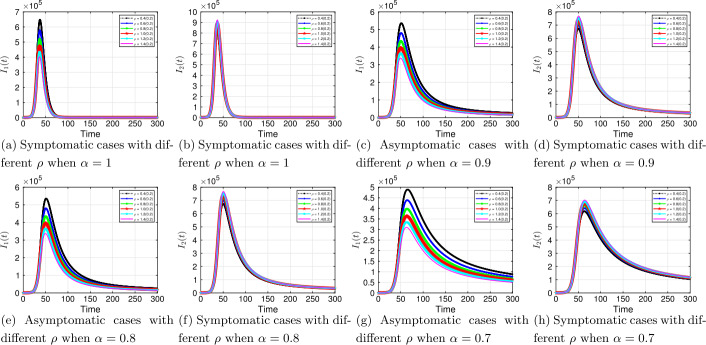
Solution profiles for the COVID-19 model with different *ρ* across different fractional order *α*

It is clear from Fig. 12 that an increase in *ρ* increases the symptomatic population, irrespective of fractional order. This is essential as it enables us to identify and isolate infected individuals from the general public and hence reducing the SARS-CoV-2 transmission.

## Summary and recommendations

The emergence of the novel coronavirus (SARS-CoV-2) has become an unprecedented threat to public health worldwide. It is now one of the greatest public health challenges in modern times with no proven cure or vaccine [70]. Many research groups are focusing on re-examining epidemic models to provide deeper mechanistic insights into the transmission dynamics and mitigation strategies of SARS-CoV-2. Here, we presented a COVID-19 model of fractional order type to explore the dynamics of the epidemic; and relied on the generalized Adams–Bashforth–Moulton method (the predictor–corrector algorithm) for fractional order to perform the numerical simulations. This method has been proven to be efficient and accurate. We investigated the effects of fractional order *α*, keeping the model parameters fixed. As illustrated in Figs. 5 to 11, a smaller fractional order reduces the peak significantly and flattens the progression curve. Modeling with fractional order provides framework that captures some important and complex features of diseases such as COVID-19.

Based on our studies from Figs. 5 to 11, we make the following recommendations:

1. Preventive measures, such as social distancing, use of face masks, and regular washing of hands, are capable in reducing *β* and are highly encouraged. In the absence of vaccine and treatment, these guidelines are optimal to contain the spread of the virus.
2. Quarantining infected individuals could be an effective control measure against the spread of coronavirus because it will reduce the value of transmission (contact) rate *β*.
3. A perfect combination of intervention measures, such as contact tracing, testing, and isolation of infected individuals, will help in the containment of the disease. Our results suggest that tracing and moving testing up has an important benefit. It reduces the number of infected individuals in the general public and thereby reduces the spread of the pandemic. Once the infected individuals are identified and isolated, the interaction between susceptible and infected individuals diminishes and transmission reduces. Furthermore, aggressive testing is also highly recommended.
4. It should be emphasized that in modeling a complex infectious disease such as the novel COVID-19, fractional order derivative is the appropriate temporal order. The behavior and pattern of spread of the coronavirus are different from country to country and city to city. It is therefore difficult (if not impossible) to model such a dynamical infectious disease with temporal order equaling 1 for different regions.

## Data Availability

Not applicable.
